# A Systematic Review of Detecting Sleep Apnea Using Deep Learning

**DOI:** 10.3390/s19224934

**Published:** 2019-11-12

**Authors:** Sheikh Shanawaz Mostafa, Fábio Mendonça, Antonio G. Ravelo-García, Fernando Morgado-Dias

**Affiliations:** 1 Instituto Superior Técnico, Universidade de Lisboa, 1049-001 Lisboa, Portugal; fabio.mendonca@tecnico.ulisboa.pt; 2 Madeira Interactive Technologies Institute, 9020-105 Funchal, Portugal; 3 Institute for Technological Development and Innovation in Communications, Universidad de Las Palmas de Gran Canaria, 35001 Las Palmas, Spain; antonio.ravelo@ulpgc.es; 4 Faculdade de Ciências Exatas e da Engenharia, Universidade da Madeira, 9000-082 Funchal, Portugal

**Keywords:** CNN, deep learning, sleep apnea, sensors for sleep apnea, RNN, deep neural network

## Abstract

Sleep apnea is a sleep related disorder that significantly affects the population. Polysomnography, the gold standard, is expensive, inaccessible, uncomfortable and an expert technician is needed to score. Numerous researchers have proposed and implemented automatic scoring processes to address these issues, based on fewer sensors and automatic classification algorithms. Deep learning is gaining higher interest due to database availability, newly developed techniques, the possibility of producing machine created features and higher computing power that allows the algorithms to achieve better performance than the shallow classifiers. Therefore, the sleep apnea research has currently gained significant interest in deep learning. The goal of this work is to analyze the published research in the last decade, providing an answer to the research questions such as how to implement the different deep networks, what kind of pre-processing or feature extraction is needed, and the advantages and disadvantages of different kinds of networks. The employed signals, sensors, databases and implementation challenges were also considered. A systematic search was conducted on five indexing services from 2008–2018. A total of 255 papers were found and 21 were selected by considering the inclusion and exclusion criteria, using the preferred reporting items for systematic reviews and meta-analyses (PRISMA) approach.

## 1. Introduction

Sleep apnea is defined by the American Academy of Sleep Medicine (AASM) [1] as a sleep related disorder characterized by the presence of breathing difficulties during sleep. The Apnea Hypopnea Index (AHI) is considered to be the most relevant metric to diagnose the existence and severity of the disorder, indicating the number of apnea events per hour of sleep. This disorder is significantly prevalent with a global estimation of 200 million people [2]. Four percent of adult men and two percent of adult women are victims of this disorder making it more common in males than in women [3]. However, among the apnea patients, 93% of middle-aged women and 82% of middle-aged men with moderate to severe sleep apnea were undiagnosed [4]. Sleep apnea can also affect the juvenile population as verified by Gislason and Benediktsdóttir [5], estimating a prevalence of three percent in pre-school children. Sleep apnea can relate to ischemic heart disease, cardiovascular disfunction, and stroke [6], daytime sleepiness [7] and can be associated with the development of type 2 diabetes [8]. In some cases, traffic accidents can occur because of drowsiness due to not sleeping well [6].

Full night polysomnography (PSG), performed in a sleep laboratory, is considered the gold standard for sleep apnea diagnosis [1]. PSG involves recording a minimum of eleven channels of various physiological signals collected from different sensors, including electroencephalogram (EEG), electrooculogram (EOG), electromyogram (EMG) and electrocardiogram (ECG), allowing researchers to achieve accurate results [9]. However, it is considered to be uncomfortable (due to a large number of wires and sensors connected to the subject’s body), expensive and unavailable to a large group of the world’s population [10]. In addition, the analysis process is time-consuming and labor-intensive [11]. Thus, it is prone to errors. Commonly, the medical facilities have a small number of professionals capable of diagnosing sleep apnea [12,13], leading to long waiting lists [14].

Various methods have been proposed in the literature to address these issues and most of them include two steps: handcraft a set of relevant features; and develop a proper classifier to provide an automatic diagnosis. These methods employ classifiers such as k-nearest neighbor (kNN) [15,16], support vector machine (SVM) [2,16,17], fuzzy logic [18,19], neural network [16,20,21], and linear discriminant analysis (LDA) [16,22]. However, these approaches have two main issues. The first one is the infinite combination of features that can be chosen, which is enhanced by the fact that combining two or more independent features, chosen as the best, cannot guarantee a better feature set [23]. However, this problem can be mitigated using proper feature selection methods and multiple algorithms have been presented in the literature: statistical estimation [9]; minimum redundancy maximum relevance (mRMR) [16]; wrapper approaches such as sequential forward selection (SFS) [15,16,24] and principal component analysis (PCA) [25]; and the genetic algorithm (GA) [21]. The second problem is the need for considerable knowledge in the specific field to create relevant features. These two issues can be solved by using deep neural networks that automatically generate features by finding patterns in the input signal from the sensor.

Although previous reviews have been performed in the field of sleep apnea detection, such as analyzing devices for home detection of obstructive sleep apnea (OSA) [26], classification methods based on respiratory and oximetry signals [26], different detection approaches [27], detection and treatment methods [28]. However, no review was previously performed to assess the current development of methods for detecting sleep apnea using deep learning. In addition to that, recent publications show a significant accuracy improvement using deep network over shallow networks. Therefore, the main focus of this review is in the analysis of such works, assessing the performance of the presented methods to provide in-depth knowledge about the applicability of deep learning in the detection of sleep apnea.

A systematic review is performed using the preferred reporting items for systematic reviews and meta-analyses (PRISMA) approach. The employed review method is presented in Section 2. The analysis of the employed signals or sensors and databases is presented in Section 3, while Section 4 presents a discussion regarding the usability and necessity of pre-processing the data. Section 5 provides a model detailed explanation of the employed classifiers that were mentioned through the review. The common performance indicators are discussed in Section 6. The key question of this review, how to implement deep learning for sleep apnea, and the comparison between different techniques are addressed in Section 7. The discussion and conclusions are presented in the final section (Section 8), with indications of the limitation and possibilities for future research in the analyzed topic. Abbreviations of different acronyms are mentioned in the Appendix A.

## 2. Materials and Methods

The review was performed considering the timeline between 2008 and 2018, based on the PRISMA style. A systematic search was conducted on Web of Science, IEEE Explorer, PubMed, ScienceDirect, and arXiv. The selected search keywords were (“sleep apnea” OR “sleep apnoea”), due to the different spellings of the word apnea, along with the AND operation and: “unsupervised feature learning”; “semi-supervised learning”; “deep belief net”; “CNN”; “convolution neural network”; “autoencoder”; “deep learning”; “recurrent neural network”; “RNN”; “long short-term memory”; “LSTM”. A total of 255 articles were found, specifically: 93 on the Web of Science; 77 on PubMed; 51 on IEEE Xplorer; 25 on ScienceDirect; 9 on arXiv. A total of 116 duplicate articles were removed from the list.

**Figure 1:**
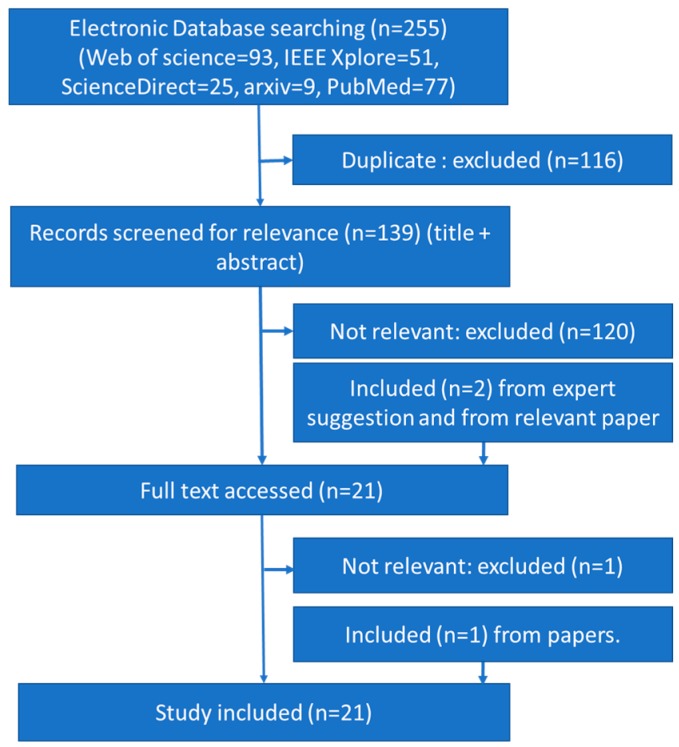
Flow chart of the process for article selection using preferred reporting items for systematic reviews and meta-analyses (PRISMA) reporting style.

The title and abstract of each article were analyzed and 19 were selected as relevant to the topic. The inclusion criteria analyzed the keywords apnea and deep network. The main exclusion criterion was non-English articles. Works that were not explicitly developed for sleep apnea detection, but could be adapted for that purpose, were also excluded. Two papers were added due to their relevance though they did not appear in the search and two were removed despite of their appearance in the search. A relevant article, found by analyzing the references of the already selected articles, was included despite not appearing in the search engines. Therefore, a total of 21 articles were selected for this review. The flow chart of the search strategy is presented in Figure 1, with n indicating the number of articles.

The last decade was chosen for this work since most of the articles (20 articles) were published in 2017 (five articles) and 2018 (15 articles). Only one was published in 2008. Therefore, within one year, the number of published articles was three times higher, highlighting the importance of this topic and the need for a review to consolidate the developed approaches and point out new research lines.

**Figure 2:**
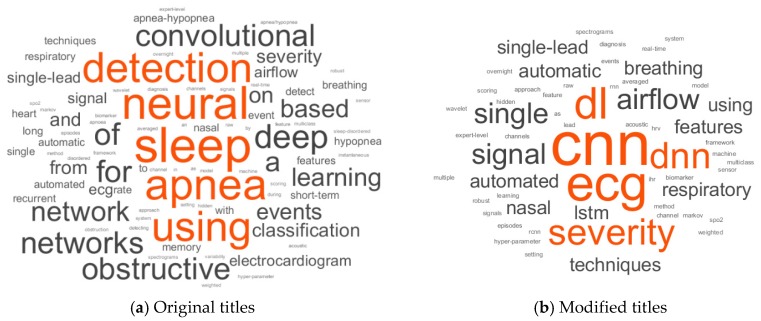
Word cloud of titles of selected papers (**a**) original titles and (**b**) modified titles. All the letters are presented in lowercase.

**Figure 3:**
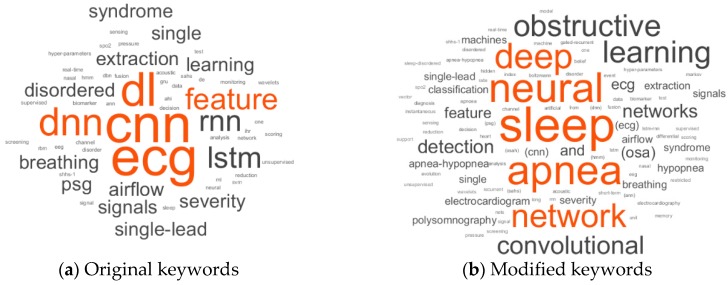
Word cloud of keywords of selected papers (**a**) original keywords and (**b**) modified keywords. All the letters are presented in lowercase.

A word cloud, presented in Figure 2a, was created from the articles’ original titles. It was challenging to understand the critical features of the implemented deep networks because of synonyms words, abbreviations and acronyms for the same word, and there were also articles and prepositions which contained no information. Therefore, a modified text with acronyms, without connecting words and the most selected words of Figure 2a, was also used to produce a word cloud presented in Figure 2b. Connecting words like using, every form of detect, classification, sleep, apnea, and events were also removed. In addition to the searched keywords for this review, a validation of the keywords selection of the papers is presented in Figure 3. From this modification and exclusion of the original text, it was possible to verify that most of the works use ECG (electrocardiography) sensors as the source signal. CNN (convolution neural network) and LSTM (long short-term memory) were the most commonly mentioned classifiers.

## 3. Signals, Sensors and Databases

The physiological signals employed to create the models can be either collected by the authors, or their respective partners or were collected before and retrieved from databases. Multiple signals and sensors can be used to detect the presence of apnea. Thus, an analysis of the most commonly used signals and sensors was performed, and the databases employed in the studies were described, providing an overview of the available tools for future researchers. A comparison summary is showed in Table 1.

### 3.1. Signal Based on Electrocardiography Sensor 

The ECG measures the electrical activity of the heart by using electrodes (the number depends on the test) that are connected to the skin, which detects small electrical changes due to depolarization and repolarization of heart muscles. The Apnea-ECG Database (AED) [29] is one of the most commonly used databases for ECG analysis. A total of 70 nighttime ECG recordings, with one-minute annotations, were provided by Philipps University, Marburg, Germany and are freely available on the PhysioNet site [30,31]. The ECG signal was sampled at 100 Hz and the recordings’ length ranges between 401 and 587 min. These ECG signals were used by Li [32], Pathinarupoth [33,34], Novak [35], Falco [36], Dey [37] and their colleagues.

Banluesombatkul et al. [38] used the Osteoporotic Fractures in Men Study (MrOS) Sleep Study (Visit 1) [39,40,41,42] Database. It has raw PSG recordings of 2911 subjects, with a minimum age of 65 years old, collected in 6 clinical centers. The ECG sampling rate was 512 Hz and a high pass filter of 0.15 Hz was used on the signal. The authors [38] had chosen 545 subjects including 364 normal subjects and 181 severe obstructive sleep apnea (OSA) subjects.

Urtnasan et al. [43] collected the full-night PSG signals of 86 subjects (65 male, 21 female) at the Sleep Center of Samsung Medical Center, Seoul, Korea (SCSMC86) [43]. The signals were collected using an Embla N7000 amplifier system (Embla System Inc., Broomfield, CO, USA) and annotated by a sleep specialist in accordance with the standards of the 2012 American Academy of Sleep Medicine (AASM) guidelines [44]. A single-lead ECG was employed with an average length of 7.4 ± 0.72 h using a sampling rate of 200 Hz. In total, 26 subjects were diagnosed with mild (5≤AHI<15) obstructive sleep apnea hypopnea (OSAH), 30 subjects with moderate (15≤AHI<30) OSAH and 30 subjects with severe (AHI≥30) OSAH. Urtnasan et al. have also collected nocturnal PSG recordings from 92 subjects (74 males and 18 females) [45] and 82 subjects (63 males and 19 females) [46] in the same sleep center (producing, respectively, the datasets SCSMC92 and SCSMC82).

### 3.2. Sensor Based on Blood Oxygen Saturation Index

The blood oxygen saturation index (SpO_2_) measures the level of oxygen in the blood. This measurement is commonly performed using a pulse oximeter that calculates the difference between the absorption of infrared and red lights to estimate the oxygen level. 

Two public databases, available at the PhysioNet web site, were used by works selected for this review, the Apnea-ECG Database (AED) [29,31] and the St. Vincent’s University Hospital/University College Dublin Sleep Apnea Database (UCD). AED had 8 recordings with a SpO_2_ signal, sampled at 50 Hz, and was used by Pathinarupothi et al. [33] and Mostafa et al. [47]. The UCD database had 25 recordings with length ranging from 5.9 to 7.7 h and sampled at 8 Hz. Cen et al. [48] and Mostafa et al. [47] used the SpO_2_ signals from the UCD database. 

Biswal et al. [49] used data from two sources, collected at the Massachusetts General Hospital (MGH) sleep laboratory, with 10,000 subjects, and from the Sleep Heart Health Study (SHHS) dataset [50], with 5804 subjects. Although the MGH dataset has five sensors, only the signals from four sensors (chest belts, abdomen belts, airflow, pulse oximetry) were used from both databases. For the MGH, the average age was 53 years old with an average total sleep time of 374.5 min, while for the SHHS, the average age and total sleep time was 63 years old and 367 min.

Choi et al. [51] collected PSG signals from 129 subjects over 20 years of age, at the Center for Sleep and Chronobiology, Seoul National University Hospital (SNUH) [51]. The signals were collected using NEUVO system (Compumedics Ltd., Victoria, Australia) and the annotation of apnea events was performed according to the 2012 AASM manual (version 2.0) [9]. A pulse oximeter was used to collect the SpO_2_ signal. 

### 3.3. Sensor Based on Sound 

The microphone is the most commonly used sensor to record breathing sounds when the subject is sleeping.

Kim et al. [52] collected full night PSG data (Embla^®^ N7000, Natus neurology) for 120 patients from the Seoul National University Bundang Hospital (SNUBH) sleep center [52]. The breathing sound was collected using a PSG-embedded microphone (SUPR-102, ShenZhen YIANDA Electronics Co. Ltd., Shenzhen, China) from a distance of 1.7 m on the ceiling above the patient’s bed. The sampling frequency of the recordings was 8 kHz. The average recording time was 7 h and 10 min.

Choi et al. [51] database SNUH, collected in Seoul National University Hospital (SNUH) [51] with 129 subjects has snoring sound collected using a microphone (previously described in Section 3.2). The sensor based on ribcage and abdomen movements apnea is the consequence of irregular breathing. Therefore, it was possible to detect irregular breathing from the rib cage and abdomen movements. 

Cen et al. [48] analyzed data from 23 subjects available at the UCD database with a combination of SpO_2_, oronasal airflow, and movements of the ribcage and abdomen.

Haidar et al. [53] used the Multi-Ethnic Study of Atherosclerosis (MESA) dataset, with 2056 full night PSG records collected by the National Sleep Research Resource (NSRR) [39], having at least 8 h of recording. The database had EEG, thoracic and abdominal respiratory inductance plethysmography, airflow (via oral or nasal thermistor and nasal pressure transducer), ECG, chin EMG, hemoglobin saturation (finger pulse oximetry), body position and leg movements. However, only the nasal airflow channel and the thoracic and abdominal channels were used, sampled at 32 Hz.

Choi et al. [51] collected abdominal volume changes using thoracic and piezoelectric sensors in the Center for Sleep and Chronobiology, Seoul National University Hospital (SNUH), from 129 subjects (previously described in Section 3.2). Biswal et al. [49] analyzed the chest and abdominal movements from 5804 subjects, chosen from the 10,000 recordings of the Massachusetts General Hospital (MGH) sleep laboratory, available in the Sleep Heart Health Study (SHHS) datasets.

The SHHS-1 dataset [54] was used by Steenkiste et al. [55], which contains data of 5804 adults with a minimum age of 40 years old. Two thousand one hundred patients (1008 females and 1092 males) with an average age of 62.5 ± 12.6 years old were chosen and the respiratory bands signal was sampled at 10 Hz [56]. They use abdores (abdomen belt placed below the lower edge of the left ribcage) and thorres (chest belts placed below left armpit) signals. ECG derived respiration signals were also used.

### 3.4. Sensor to Detect Airflow 

The airflow (AF) signal from the MrOS sleep database (Visit 2) [39,40,41,42] was used by Lakhan et al. [57]. In total, 1026 men with a minimum age of 65 years old were enrolled in sleep examinations at six clinical centers. Similarly, to MrOS (Visit 1), raw PSG signals were collected in European data format (EDF) files with XML annotation files. The AF signals were recorded with ProTech Thermistor sensors using a 32 Hz sampling rate. The authors randomly selected 520 subjects from the whole database to do the analysis.

Multiple pressure changes occurred during the breathing process and were measured by a cannula transducer [58]. The PTAF 2 (Pro-Tech, Woodinville, WA, USA) for measuring nasal pressure was used by Choi et al. [51] to record the breathing signal of 129 subjects. It was the same format as the Center for Sleep and Chronobiology, Seoul National University Hospital (SNUH) [51] (previously described in Section 3.2).

Biswal et al. [49] analyzed the airflow signals from Massachusetts General Hospital (MGH) sleep laboratory, with 10,000 subjects, and from Sleep Heart Health Study (SHHS) datasets, with 5804 subjects. The nasal airflow signals, recorded with a sampling rate of 32 Hz, from MESA dataset [39] were used by McCloskey et a. [59] and Haidar et al. [53,60].

Cen et al. [48] used oronasal airflow from 23 UCD [61] database recordings. 

## 4. Data Pre-Processing 

### 4.1. Raw Input Signal 

The unprocessed signals (raw signals) can be directly employed as the input of the classifier as proposed by Mostafa et al. [47] using raw SpO_2_ signal from two databases, by resampling the signal, at 1 Hz, to provide a uniform dataset. The raw airflow, respiration (chest and abdomen belts) and SaO2 signals were used as inputs for a CNN by Biswal et al. [49]. Haidar et al. [53] used three raw respiratory channels of PSG recordings (nasal airflow, thoracic and abdominal plethysmography) with normalization based on the mean (μ) and standard deviation (σ) of the normal samples for each subject and the type of channel [53].

(1) $$S_{x}{,}_{t}=\frac{s_{x}{,}_{t}-\mu_{s_{n}{,}_{x}{,}_{t}}}{\sigma_{s_{n}{,}_{x}{,}_{t}}}$$

where $s_{x}{,}_{t}$ is the raw signal for subject x, t is the signal type (either nasal, thoracic or abdominal plethysmography) and n is total number of normal samples of the subject x.

Cen et al. [48] combined three signals (SpO_2_, oronasal airflow and ribcage and abdomen movements), $N_{channels}=3$, with a sampling frequency of $F_{s}\;\mathrm{of}\;16\;\mathrm{Hz}$ and a 5-s window length, $\Delta_{w}$. Therefore, the number of samples was

(2) $$N_{samples}=\Delta_{w}\times F_{s}\times N_{channels}=240$$

These samples were reshaped into a 16 × 15 matrix and padded with zeros to get a square 16 × 16 matrix.

### 4.2. Filtered Signal

Commonly, the raw signal is contaminated with noise that can significantly affect the classifier’s performance. The employment of filters can mitigate this issue. For the ECG signal, the undesired noise can be removed by applying a bandpass filter (5–11 Hz) on the raw signals [43,44,45,46]. A notch filter at 60 Hz and a bandpass second-order Butterworth filter, with cutoff frequencies at 5 and 35 Hz, can also be used to clean the ECG signal [38]. 

Kim et al. [52] removed the noise from the breathing sound using a two-stage filtering process: first, a spectral subtraction filtering method [63] was employed to improve the efficiency [64]; a sleep stage filtering was used to eliminate the noises originating from conversations and the sound of duvet [52]. 

Steenkiste et al. [55] used a fourth-order low-pass zero-phase-shift Butterworth filter, with a cut-off frequency of 0.7 Hz, to reduce the noise in the respiratory signals [65]. The motion artifacts and baseline wander were removed by performing a subtraction of a moving average filtered signal, with 4 s width, to the original signal.

Denoising of the AF signals can be performed by applying a low-pass filter, with a 3 Hz cut-off, [57]. Choi et al. [51] down-sampled the nasal pressure signal to 16 Hz and for reducing baseline drifts and high frequency noise fifth-order infinite impulse response (IIR) filter, with 0.01 Hz (high-pass), and 3 Hz (low-pass) was used. An adaptive normalization method [66] was also applied to keep the part where the amplitude of respiration is small. The employed normalization $F_{Norm}$ was defined by [51].

(3) $$F_{Norm}\left(k\right)=min\{0.95F_{Norm}\left(k-1\right)+0.05A\left(k\right),0.95F_{Norm}\left(k-1\right)+0.05\sigma \left(k\right)$$

(4) $$\mathrm{where}\;A\left(k\right)=\frac{1}{f_{s}}\sum_{i=k*f_{s}}^{\left(k+1\right)*f_{s}-1}abs\left(x\left(i\right)\right)$$

(5) $$\mathrm{And}\;\sigma \left(k\right)=\sqrt{\frac{1}{f_{s}-1}\sum_{i=k*f_{s}}^{\left(k+1\right)*f_{s}-1}{\left(x\left(i\right)-\bar{x}\left(k\right)\right)}^{2}}$$

This normalization was repeated for each second considering $\bar{x}\left(k\right)$ as the average value of the signal in one second and $f_{s}$ as the number of samples. 

### 4.3. Spectrogram

Biswal et al. [49] have used the signal’s spectrogram as the input calculating the power spectral density (PSD) using Thomson’s multitaper method. For EEG and EMG, the window size of PSD was 2 s, increasing to 30 s for the respiration signals. McCloskey et al. [59] calculated spectrograms of the nasal airflow signal by using continuous wavelet transform (CWT) with the analytical Morlet wavelet. Frequency axes of the spectrogram images were scaled by log2 to show high frequency features with a similar size to the low frequency features.

### 4.4. Heart Rate from ECG

The ECG inter-beat intervals (RR-ECG) or instantaneous heart rates (IHR) (R to R interval from ECG) instantaneous heart rates (IHR) can be defined as:

(6) RR(i)=R(i+1)−R(i), i=1,2,…, n−1

Pan-Tompkins [67] developed an algorithm to detect these intervals and it was used by Novak et al. [35] and Li et al. [32]. Li et al. [32] used the median filter proposed by Chen et al. [68] to remove physiologically uninterpretable points and interpolate the RR interval series into 100 points to have a uniform length. Cheng et al. [62] employed the RR series analysis adjusting the ECG recordings to a 240 × 240 matrix.

An alternative metric, named beats per minute (bpm), was employed by Pathinarupothi et al. [33] and it can be calculated using:

(7) $$HR\left(t\right)=\frac{60}{RR}\mathrm{bpm}$$

Pathinarupothi et al. [33] used the Physionet WFDB toolkit [69] to derive the IHR series from the ECG signals. To keep the size of the input constant, the system’s first 30 IHR values of each annotated minute were chosen for IHR signals and a constant length vector of 60 was chosen for the SpO_2_ signal by the authors. In another work, Pathinarupothi et al. [34] also calculated the IHR series using Physionet WFDB toolkit [69] and it was converted to a 60 beats length input.

### 4.5. Features 

I. De Falco et al. [36] used twelve typical heart rate variability (HRV) parameters from the ECG based HRV, related to the frequency domain, the time domain, and the non-linear domain, that were created using Kubios [70] developed at the University of Kuopio, Finland [36]. D. Novak et al. [35] also extracted features related to the frequency domain and the time domain from ECG based heart rate. P. Lakhan et al. [57] extracted 17 features from overnight AF signals and used it as the input of the classifier.

## 5. Classifiers

In a broader sense there were three main types of deep networks used by the authors: deep vanilla neural network (DVNN), convolution neural network (CNN) and recurrent neural network (RNN). 

### 5.1. Deep Vanilla Neural Network (DVNN)

There are deep networks with the final structure resembling classical neural networks with more than one hidden layer. However, sometimes, these classifiers train strategy and layer construction are a little bit different than the classical one. These types of classifiers are mentioned in this work as deep vanilla neural networks (DVNNs). Mainly, three types of DVNN were employed by the authors of the reviewed works: the multiple hidden layers neural network (MHLNN); stacked sparse autoencoders; and deep belief networks.

#### 5.1.1. Multiple Hidden Layers Neural Networks

A feedforward neural network inspired by biological neurons does not have a loop or cycle and each neuron in one layer has directed connections to the neurons of the subsequent layer. The output of the previous layer $X_{n-1}$ is multiplied with a weight $w_{n}$, pass through an activation function φ and a bias $b_{n}$ is added [71]. Thus, the output of the neuron i is given by:

(8) $$\vartheta_{i}=\varphi \left(\sum_{j=1}^{n}w_{n}X_{n-1,j}+b_{n}\right)$$

The layers between the input and output layers are named hidden layers. A typical example of a deep learning model is the feedforward deep network, or multilayer perceptron [72]. A feedforward neural network with more than one hidden layer can be considered as a deep network. In this work, a classical neural network with multiple hidden layers is indicated as multiple hidden layers neural network (MHLNN).

#### 5.1.2. Deep Stacked Sparse Autoencoder 

A deep autoencoder is composed of several stacked encoder layers that can apply a sparsity regularization forming the deep sparse autoencoder (SpAE). An autoencoder is composed of an encoder and a decoder and these networks are trained with the goal of minimizing the cost function between the input and the output through an unsupervised method. The cost function usually measures the error between the input x and the output $\hat{x}$. The encoded output is expressed by:

(9) $$z=\varphi_{1}\left(W_{1}x+b_{1}\right)$$

and the output of the decoder is

(10) $$\hat{x}=\varphi_{2}\left(W_{2}z+b_{2}\right)$$

where $\varphi_{1}$ and $\varphi_{2}$, $W_{1}$ and $W_{2}$, $b_{1}$ and $b_{2}$ are, respectively, the transfer function, weight and bias of the network encoder and the decoder.

A sparsity regularization factor is added to the cost function to produce the sparse autoencoders [73]. If the average activation of a unit is [32]:

(11) $$\hat{p_{i}}=\frac{1}{n}\sum_{j=1}^{n}\varphi \left(w_{i}x_{j}+b_{i}\right)$$

Then the sparsity ($\Omega_{sparsity}$) can be implemented by adding a regularization term that takes a large value when the average activation value, $\hat{p_{i}}$ of a neuron i and its desired value, p, are not close in value [73]. It is frequently calculated using Kullback–Leibler divergence (KL):

(12) $$\Omega_{sparsity}=\sum_{i=1}^{n_{h}}KL(p||\hat{p_{i}})=\sum_{i=1}^{n_{h}}p\;log\left(\frac{p}{\hat{p_{i}}}\right)+\left(1-p\right)log\left(\frac{1-p}{1-\hat{p_{i}}}\right)$$

where $n_{h}$ is the number of hidden neurons in the network.

A supervised learning process is performed at the end of the unsupervised learning to finetune the weights of the network. 

#### 5.1.3. Deep Belief Network

Deep belief networks (DBN) are probabilistic generative models that are composed of multiple layers of hidden variables. The hidden layers are composed by restricted Boltzmann machines (RBM), an undirected bipartite graph, and the output layer perform the classification [74].

The training procedure is like the process employed in the stacked autoencoder (SAE) using unsupervised learning to individually train each hidden layer and afterward use supervised learning to finetune the weights. Therefore, the DBN model can be expressed by:

(13) $$P\left(x,k_{1},k_{2},\ldots ,k_{l}\right)=P\left(x|k_{1}\right)P\left(k_{1}|k_{2}\right)\ldots P\left(k_{l-1}|k_{l}\right)$$

where each [74] $P\left(k_{l-1}|k_{l}\right)$ is an RBM. The conditional distribution on the hidden units K and the input X can be given by logistic functions [74]:

(14) $$P\left(X=1|,K\right)=\varphi_{e}\left(wk+b\right)$$

where $\varphi_{e}\left(a\right)=1/\left(1+exp\left(a\right)\right)$, w is the weight and b is the bias.

### 5.2. Convolution Neural Network

CNNs are commonly composed by combinations of five different types of layers: input; convolution; activation functions such as rectified linear units (ReLU); pooling or sub sampling; classification (commonly a fully connected layer with the softmax function). There are also batch normalization and dropout layers that can be added to CNN.

The network produces features using different convolution kernels of convolution layers [72]. The values of the kernels are changed during the training phase for a specific task [75]. If the whole convolution layer is considered, the feature maps can be seen as a n+1 dimension map where n is the dimension of the input [76]. The equation for the feature map of the 3D convolution layer is:

(15) $$C_{d}=\varphi_{r}\left(k_{d}⊛f+b_{d}\right)$$

where $1\leq d<nk_{d}$, $nk_{d}$ is the number of convolution kernels in a layer, C is the feature map of the entire convolution layer ($C\in {\mathbb{R}}^{i\times j\times nk_{d}}$), ⊛ is the n dimensional convolution operation, k is the kernel, f is the input matrix for the first layer it could be the data x, b is the bias and $\varphi_{r}$ is the non-linear activation function. The most popular non-linear activation function is the ReLU given by:

(16) $$\varphi_{r}\left(z\right)=\{\begin{array}{l} 0,\;z<0\\ z,\;z\geq 0 \end{array}$$

For dimensionality reduction, CNN uses the pooling or sub-sampling layers. Down sampling the signal from the previous layer reduces the artifacts and sharp variations [77]. The polling operation commonly outputs either the maximum value (maximum pooling) or the average value (average pooling) of the kernels. Therefore, the pooling layer output $P_{d}$ can be expressed by:

(17) $$P_{d}=\varphi_{p}\left(\sigma_{pool}\left(f_{d}\right)\right)$$

where $f_{d}$ represents the intermediate feature maps and d is the number of pooling filters in the layer. The pooling layer can have its own activation function $\varphi_{p}$ or not depending on the designer. 

The fully-connected layer produces the output Y which is the output of the activation function [71,78]:

(18) $$Y_{\;}=\varphi_{f}\left(\sum_{j=1}^{n}\;f_{j}\times w_{j}+b\right)$$

where f is the input (feature maps coming from previous layer), n the number of inputs, w the weight and $\varphi_{f}$ could be any function chosen by the designer. Commonly, in last layer, it is the softmax function, $\varphi_{softmax}$, which is also known as the normalized exponential function [79]. It can be used to represent a categorical distribution where the input of the function is z that is a probability distribution over k different possible outcomes:

(19) $$\varphi_{softmax}\left(z^{\left(i\right)}\right)=\frac{e^{z^{\left(i\right)}}}{\sum_{j=0}^{k}e^{z_{k}^{\left(i\right)}}}$$

A batch normalization layer allows us to reduce the internal covariate shift of the network [80]. This layer normalizes its inputs $z_{i}$ over a mini batch $\beta =\left\{z_{1},z_{2},\ldots ,z_{m}\right\}$ by first calculating the mean $\mu_{B}$ and variance $\sigma_{B}^{2}$ over a mini batch and over each input channel. Then, it calculates the normalized activations as:

(20) $$\hat{z_{i}}=\frac{z_{i}-\mu_{B}}{\sqrt{\sigma_{B}^{2}+\epsilon }}$$

(21) $$\mu_{B}=\frac{1}{m}\sum_{i=1}^{m}z_{i}$$

(22) $$\sigma_{B}^{2}=\frac{1}{m}\sum_{i=1}^{m}{\left(z_{i}-\mu_{B}\right)}^{2}$$

where ϵ improves numerical stability when the mini-batch variance is very small.

A dropout layer can be used to prevent overfitting by setting the input elements to zero with a given probability [81,82].

### 5.3. Recurrent Neural Network

RNNs are neural networks with recurrent connections where the current value of the hidden node output $h_{t}$ is updated according to the previous unit $h_{t-1}$ and current input $x_{t}$ as [83]:

(23) $$h_{t}=\varphi \left(W^{x,h}x_{t}+W^{h,h}h_{t-1}+b\right)$$

Two types of RNN were employed by the reviewed works; the long short-term memory (LSTM) and the gated recurrent unit (GRU).

#### 5.3.1. Long Short-Term Memory

LSTM network allows time steps to be passed further compare with a simple RNN [45,55,84]. The memory cell extension of this network facilitates the process of learning [45]. Each memory cell contains three main gates: an input gate (ig), an output gate (og) and a forget gate (fg) [45]. If the gates are represented as vectors, they have the same size as the hidden value vector (h), $c_{t}$ represents the cell state, $\varphi_{nl}$ and $\varphi_{\tau }$ denotes the nonlinear and hyperbolic tangent functions. The input gate controls the flow of input activations into the memory cell by:

(24) $$ig_{t}=\varphi_{nl}\left(W^{x,ig}x_{t}+W^{h,ig}h_{t-1}+b_{ig}\right)$$

The output gate controls the output flow of the cell activations into the rest of the network considering:

(25) $$og_{t}=\varphi_{nl}\left(W^{x,og}x_{t}+W^{h,og}h_{t-1}+b_{og}\right)$$

The forget gate scales the internal state of the cell before adding it as input through the self-recurrent connection of the cell. Therefore, it adaptively forgets or resets the cell’s memory.

(26) $$fg_{t}=\varphi_{nl}\left(W^{x,fg}x_{t}+W^{h,fg}h_{t-1}+b_{fg}\right)$$

(27) $$g_{t}=\varphi_{nl}\left(W^{x,c}x_{t}+W^{h,c}h_{t-1}+b_{c}\right)$$

(28) $$c_{t}=f_{t}*c_{t-1}+i_{t}*g_{t}$$

(29) $$h_{t}=o_{t}*\varphi_{\tau }\left(c_{t}\right)$$

#### 5.3.2. Gated Recurrent Unit

A gated recurrent unit GRU is a modified version of the LSTM [85,86]. It uses an update gate $\;\left(ug_{t}\right)$ instead of a forget gate and an input gate. Also, this networks does not have separate memory cells [83]. If $rg_{t}$ represents reset gate and $\varphi_{nl}$ and $\varphi_{\tau }$ represents nonlinear and hyperbolic tangent functions [83]:

(30) $$ug_{t}=\varphi_{nl}\left(W^{x,ug}x_{t}+W^{h,ug}h_{t-1}+b_{ug}\right)$$

(31) $$rg_{t}=\varphi_{nl}\left(W^{x,rg}x_{t}+W^{h,rg}h_{t-1}+b_{rg}\right)$$

(32) $$h_{t}^{\prime }=\varphi_{\tau }\left(Wx_{t}+Wh_{t-1}*rg_{t}\right)$$

(33) $$h_{t}=\left(1-ug_{t}\right)*h_{t-1}+z_{t}*h_{t}^{\prime }$$

## 6. Performance Indicators

Multiple metrics can be used to assess the performance of the classification. The most common parameters shared among all the works are calculated by considering the true positive (TP), true negative (TN), false positive (FP) and false negative (FN) values. These parameters can be expressed as defined by Baratloo et al. [87] where TP is the number of cases correctly identified with the disorder(/patients/apnea), FP is the number of cases incorrectly identified with the disorder, TN is the number of cases correctly identified as normal(/healthy/ non-apnea) and FN is the number of cases incorrectly identified as normal. However, an interchangeable definition of TP and FP was used in some of the reviewed works [43,46]. It is possible to define the accuracy (Acc), specificity (Spc), precision or positive predictive value (PPV) and recall or sensitivity (Sen) as:

(34) $$Acc=\frac{\left(TP+TN\right)}{\left(TP+TN+FP+FN\right)}$$

(35) $$Spc=\frac{TN}{TN+FP}$$

(36) $$PPV=\frac{TP}{\left(TP+FP\right)}$$

(37) $$Sen=\frac{TP}{\left(TP+FN\right)}$$

For binary classifiers (models with only two possible outputs), recall has the same definition as Sen. However, these metrics can be strongly affected by imbalanced classes in the dataset. Other metrics are used to address this issue such as a combined objective (*CO*):

(38) $$CO=\frac{1}{3}\left(Acc+Sen+Spc\right)$$

and the area under the receiver operating characteristic curve (AUC). The receiver operating characteristic curve can be created by considering the true positive rate (TPR) versus the false positive rate (FPR) with different thresholds for the classifier [88]. Then the area under the curve is calculated to determine the AUC values. An alternative metric is the $F_{1}$ score, given by:

(39) $$F_{1}=2\frac{PPV*Sen}{PPV+Sen}$$

A weighted proportion, $w_{i}$, can be introduce to the $F_{1}$ producing:

(40) $$F_{1w}=\sum_{i}2.w_{i}\frac{PPV_{i}*Sen_{i}}{PPV_{i}+Sen_{i}}$$

where $w_{i}=n_{i}/N$ i is the class index, N is the total number, $n_{i}$ is the number of classes i.

Other ways of solving the imbalance could be down-sampling or up-sampling. A balanced bootstrapping is also proposed and used [55]. A comprehensive review of learning from the imbalanced dataset [89], handling the problem [90], and used technique in deep learning [91] was discussed in the literature.

## 7. Implementation of Classifiers and Performance

### 7.1. Deep Vanilla Neural Network

On the reviewed articles the DVNN was employed using either automatic [32,47] or human crafted feature learning [36,52,57].

#### 7.1.1. Automatic Feature Learning Using DVNN

A hidden Markov model with autoencoder was used by Li at el. [32] using automatic feature learning. The implementation used 100 points of the RR series, selected by the Pan-Tompkins algorithm [67] which were passed through a median filter [68] as an input. A SAE was used for classification and the data was divided into a 50% training set (35 subjects) and a 50% test set (35 subjects). The training process was based on the mixture of unsupervised learning with finetuning at the end. First, a single hidden layer SAE unsupervised training was done for primary feature extraction then it was fine-tuned by using a logistic regression layer. After that, these extracted features were used as the corresponding observation vector $\left(O_{t}\right)$ of a Markov model [92] which belonged to two Markov states $S=\left\{S_{N},S_{A}\right\}$ where $S_{N}$ is the normal and $S_{A}$ the apnea state. Then a soft decision fusion of two separate classifiers (ANN, SVM) was done based on the confidence score maximization strategy that considered the classifier quality information [93]. Two deep network structures were analyzed and the highest accuracy (83.8%) was achieved using 100 neurons on the first hidden layer (HL) and the second HL with 10 neurons.

A DBF with two HL was analyzed by Mostafa et al. [47] using the SpO_2_ signal resampled at 1Hz with tenfold cross validation. It was verified that the selected number of neurons had a significant impact on the results. Therefore, a grid search approach was employed, varying the number of neurons from 30 to 180, with intervals of 30 neurons, in two hidden layer DBN. The optimum number of hidden neurons (90 in the first HL and 60 in the second HL) was found by maximizing the CO (Equation (38)). The achieved accuracy for the UCD [61] and AED [29] databases was, respectively, 85.26% and 97.64%.

#### 7.1.2. Human Crafted Feature Learning Using DVNN

Breathing sounds during sleep were analyzed by Kim et al. [52] using a MHLNN with two hidden layers (first with 50, and second with 25 nodes) and two dropout layers with four classes (normal, mild, moderate and severe). Using tenfold cross validation, windows of 2.5, 5, 7.5 and 10 s were tested, and five seconds achieved better performance. A patient wise classification was performed, with an average global accuracy of around 75% by the MHLNN which slightly less than the performance attained by both SVM and logistics classifier.

Lakhan et al. [57] produced 17 features from AF signal and a fully-connected neural network with layers size of 1024, 512, 256, 128, 64, 32, 16, 8, and 4 hidden nodes with a softmax function at the end. Average Acc of 83.46%, 85.39% and 92.69% were achieved using tenfold cross validation for three cutoff points of the AHI (5, 16 and 30) respectively.

Falco et al. [36] used evolutionary algorithms (EAs) with a data subsampling technique (the training set consisted of 60% and the test set consisted of 40% of the data) to reduce the simulation time to find the best hyperparameter of the MHLNN. The HRV was calculated from the twelve typical parameters (features) of HRV related to the frequency domain, the time domain, and the non-linear domain, which were extracted from the one-minute segment. It was verified that 2 HLs with 23 and 24 hidden units using ReLU as an activation function produced the highest accuracy (68.37%).

### 7.2. Convolutional Neural Network (CNN)

CNN was mainly developed to classify images. However, some authors [37,43,46,51,60] adapted the concept by employing a one dimensional CNN (CNN1D) network for signal classification. Haider et al. [53] used three one dimensional signals hence producing a CNN1D with three channel inputs. Other authors [48,59] converted the one dimensional signal to a two dimensional input to employ the two dimensional CNN (CNN2D) network directly. An analysis of both CNN1D and CNN2D was performed by McCloskey et al. [59] to assess performance.

#### 7.2.1. CNN1D

The signal from a single-lead ECG was analyzed by Urtnasan et al. [46] using a CNN1D with an hold-out method (training set had 63 subjects, test set had 19 subjects). The signal was segmented into 10 s intervals, unlike one minute segments performed by other authors [32,47], each having 2000 sample points. The network was composed of different sizes of convolution, activation, and pooling layers, followed by dropout. The input signal was normalized by batch normalization and a ReLU was employed as an activation function. Following that, batch normalization and a ReLU layer, and a set of convolution and pooling layers was repeated. At the end, a dropout layer followed by a fully connected layer and a softmax activation function was used for binary classification. In between the final layer and the batch normalization layer, the set of layers was repeated. Seven CNN models with a number of layers varying from three to nine, with a one-layer increment, were studied. The highest accuracy (96%) was achieved using the CNN with six layers using the $F_{1}$ score as a defining parameter.

Urtnasan et al. [43] also used the CNN1D for multiclass classification (normal, apnea and hyperpnea). The input of the network was 10 s long contained and 2000 samples. A hold-out method was used to test the model similar to what was done in the previous work [46]. The network architecture included batch normalization (batchnorm), convolution (conv1D), maximum pooling (maxpool), dropout and fully connected layers. The first layer was batchnorm followed by conv1D (20 filters with [50×1]) and maxpool ([2×1]). Afterward, a set of variously sized conv1D, maxpool and dropout (p=0.25) was repeated and stacked, one after another until the final softmax layer. The six-layer CNN achieved 90.8% mean accuracy among the classes.

Dey et al. [37] also employed a CNN1D to analyze one minute segments of a single lead ECG signal, each with 6000 samples. Unlike other implementations, it used only convolution and fully connected layers. The pooling was performed using convolutional pooling. Authors tested the model with different training:test dataset ratios from 50:50 to 20:80 where 50:50 had the best average accuracy of 98.91%.

Binary classification (either apnea or normal) based on the nasal airflow analysis was performed by Haidar et al. [60] with a CNN1D classifier and a balanced dataset. The network consisted of three convolutional layers, each had 30 filters with [5×1] kernel size, five strides, each followed by a max pooling layer with [2×1], and one fully connected layer with a soft-max activation function. It had two output nodes for each class (normal or abnormal). The activation function ReLU was chosen because of the best accuracy and fastest training time by [60] by evaluating other activation functions. The model achieved an average accuracy of 75%.

The signal from a single-channel nasal pressure was analyzed with a CNN by Choi et al. [51] to detect one second apnea events. The database was divided into training (50 subjects), validation (25 subjects) and testing (104 subjects). It was tested using the class balance hold-out method. Overlapping windows with length ranging from five to 10 s were tested and multiple configurations of the network were analyzed, changing the number of convolution layers (one to three), the number of convolution filters (5,15,30), the kernel sizes for convolutions (4,8,16,32) the strides for convolutions (1, 2, 4, 8, 16) and the strides for pooling (1,2). It was verified that a 10 s windows with three convolution layers, two maxpooling layers and two fully connected layers achieved the highest accuracy (96.6%).

A CNN1D with three input signals was tested by Haider et al. [53], analyzing the nasal flow, the abdominal and thoracic plethysmography signals using hold-out methods with 75% training and 25% test datasets. Two back to back convolution layers with a subsampling layer (conv-conv- maxpooling) in a three-cascading state with a final layer of a fully connected layer were studied. It was verified that the performance of the model with three channels was better than any single or double channels model, with an average accuracy of 83.5%.

McCloskey et al. [59] have also performed a multiclass classification(normal, apnea and hyperpnea), by analyzing the nasal airflow signal, normalized with 30 s epochs, with an input size of 960 samples. Three sets of conv-conv-maxpooling layers followed by one fully connected layer made the CNN1D. The first convolution layer in the set had 32 filters with a kernel size of [3×1], stride of three and ReLU as an activation function. The second convolution layer also had ReLU as an activation function with a kernel size of [2×1], a stride of two. The maxpooling layer kernel was [2×1] with a stride of two. The output had three nodes representing three classes. The CNN1D achieved an average accuracy of 77.6%.

#### 7.2.2. CNN2D

The spectrogram of the nasal airflow signal, calculated by using continuous wavelet transform (CWT) with the analytical Morlet wavelet, was fed to a CNN2D by S. McCloskey et al. [59]. The network had two convolutional layers with ReLU activation layers afterward and one 2-D max pooling layers followed by a fully connected layer and a softmax layer with three output nodes representing the three classes (normal, apnea and hyperpnea). The model achieved an average accuracy of 79.8%.

Chen et al. [48] used CNN2D with leave one out cross validation, which has three input signals (blood oxygen saturation, oronasal airflow, and ribcage and abdomen movements) with one second annotation. A two-dimensional matrix with zero padding was created as input to the network that consisted of two convolution layers, two subsampling layers and a fully-connected layer connected to the output layer with three nodes. The multiclass classification overall accuracy was 79.61%.

### 7.3. Recurrent Neural Network (RNN)

SpO_2_ and IHR signals were tested by Pathinarupothi et al. [33] as an input to as LSTM. The dataset was divided into 50% for training, 40% for testing and 10% for validation. With only the SpO_2_ signal, the single layer, 32-memory block, LSTM and the 32-memory block stacked LSTM achieved an AUC of 0.98. With only the IHR signal the 32-memory block stacked LSTM achieved a 0.99 AUC for severity detection (apnea or non-apnea). Combining both signals provided a 0.99 AUC in both single layers and stacked LSTM.

The same authors [33] also used IHR for apnea and arrhythmia classification with higher accuracy and F1 score of 1 [34] using a fivefold cross-validation technique. Both the single layer and the stacked layers LSTM (two layers) were tested and it was verified that better results were attained by the two-layer stacked LSTM. However, the single layer and 32 memory cells work better than two-layer stacked LSTM-RNN model.

To capture temporal information and accurately model the data Steenkiste et al. [55] used a LSTM [85] neural network. Balanced bootstrapping was employed to balance the dataset, where the entire minority class was used each time with an equal size of the majority class. These balanced datasets were used for each LSTM model which had one LSTM layer with three dropout layers and ends with an output layer. In the end, the probabilities of the LSTM models were aggregated into a single probability prediction per epoch by averaging. An averaged probability greater or equal to 50% was used to determine the presence of apnea. The authors also used the same LSTM network structure with human-engineered time-domain and the frequency-domain features instead of raw respiratory signals [55]. Because it used features with LSTM it is denoted as FLSTM. A performance valuation was also done with three signals respiratory signals (abdores, thorres and EDR) with non-temporal models with temporal models. Both temporal models (FLSTM, LSTM) did better than the non-temporal models (ANN, logistic regression (LR), random forest (RF)). Among the temporal models, LSTM did better than FLSTM in all three signals (Table 2). Though in the original paper, the authors detected apnea severity in this review, it was not included because the presentation of severity was different compared with other work (for severity please check Figure 7, Figure 8 and Figure 9 of the original work [55], in addition, it was quite difficult to calculate the exact values from the figures).

A three layered FLSTM was used by Novak et al. [35] to calculate apnea events using heart rate variability with features as input. The hidden layers of the network contained five blocks, each consisting of seven memory cells, achieving an average accuracy of 82.1%.

Cheng et al. [62] employed a four layered LSTM to detect OSA using 20 subjects for train and 10 subjects for test and the RR-ECG signal. The network consisted of a recurrent layer and a data normalization layer, repeated four times, followed by a softmax layer, achieving an average accuracy of 97.80%.

Urtnasan et al. [45] used the normalized ECG signal with 74 subjects for training and 18 subjects for testing and six RNN layers were used to form an LSTM and a GRU. The $F_{w}$ score of the LSTM and GRU was, respectively, 98.0% and 99.0%.

### 7.4. Combination of Multiple Deep Networks

A combined deep recurrent and convolutional neural networks (RCNN) was evaluated by Biswal et al. [49], using airflow, SaO2, chest and abdomen, belts signals to determine the AHI. A hold-out method with 90% of data for tanning and 10% of data for testing was used. Both waveform representation and spectrogram representation were employed as input signals for a CNN and a combination of CNN and RNN (RCNN). The RCNN with spectrogram representation achieved the highest accuracy (88.2% in MGH and 80.2% in SHHS).

A different approach was presented by Banluesombatkul et al. [38], achieving 79.45% of global accuracy (detecting extremely severe OSA subjects from normal subjects) by combining CNN1D, LSTMs and MHLNN (in original work it was defined as deep neural network (DNN)) to detect sleep apnea from 15 s window using a tenfold cross validation method. This structure was used for automatic extraction of the features using the CNN1D with 256, 128 and 64 units, where each convolution layer was followed by a batch normalization layer and ReLU was used as an activation function. Then a LSTM, with 128, 128, and 64 units, respectively, and a recurrent dropout of 0.4, was then stacked to extract temporal information. At the end of the network, a MHLNN (with fully connected layers) was stacked with layers of size 128, 64, 32, 16, 8, and 4 hidden nodes followed by a SoftMax function for the classification.

## 8. Discussion and Conclusions

This systematic literature review has synthesized and summarized the published deep classification methods for sleep apnea detection. From the selected 21 studies, the main findings are provided below.

It was verified that a significant number of papers were published in the last two years, indicating a strong interest in the research community on this topic. Comparison between the deep networks and parameter choice of the deep network is still a matter of ongoing research and a very hot topic. In addition to that, which sensor or signal is best for the apnea detection is still in question.

The ECG sensor based signal was the most commonly used, which could be justified as indicated by Mendonça et al. [27], that for a single source sensor, ECG signals provided the highest global classification. However, sleep apnea is directly related to respiration. Thus, this higher accuracy with ECG signals could happen due to the use of public datasets that are less affected by noise [27]. For the works based on a single sensor, Pathinarupothi et al. [33] achieved the best results using the SpO_2_ signal comparing IHR from ECG. Therefore, the universality of better ECG signals performance is not true. However, a direct comparison between different works between the different signals performance parameters is not fair for this review because of the use of different classifiers and different databases.

It was verified that using more than one signal from sensors improves the predictive capability of the models as reported by Haidar et al. [53]. This is understandable because the gold standard of sleep apnea tests uses several signals. However, the main research goal of most of the work is to achieve a respectable result using fewer sensors.

Most of the work with deep networks outperformed the shallow networks except for the work of T. Kim et al. [52]. In their work, a deep network performed slightly less than the shallow network. However, they used deep network with human engineered features. Similar kinds of work where authors [57] used features with deep network MHLNN outperformed classical machine learning techniques. Therefore, for the work of T. Kim et al. [52] may be a feature selection process or hyperparameter choice of the deep network.

CNN was the more commonly used classifier and approach based on both CNN1D and CNN2D as was presented. However, it was not possible to indicate what this is the best type of network since the testing conditions were different in all works. However, McCloskey et al. [59] compared both and verified that 2-D spectrogram images of the nasal airflow performed better than raw 1-D signal with CNN. A similar conclusion was attained by Biswal et al. [49] where RCNN with spectrogram representation achieved a higher accuracy. Analyzing the three works of Urtnasan et al. using CNN1D [43,46] and RNN [45] where they had collected the data from the same hospital, it was possible to verify that RNN outperformed the CNN. However, more research is needed to reach a definitive conclusion. The same type of conclusion can be achieved by analyzing the works that have employed LSTM and GRU.

Hyperparameters optimization is also a problem in deep network implementation. Some works [43,46,47] have verified that just blindly increasing the number of layers or neurons in the hidden layers did not increase the performance. Most of the works chose the hyperparameters with an educated guess or by trial and error methods. Others used a predefined search space and tried to find a best solution [43,46,47]. A possible alternative solution was presented by Falco et al. [36], were an EA was used to choose the hypermeters.

For performance purposes, dominating methodologies were hold-out and cross-validation methods. Hold-out does not test all the dataset. It is understandable that due to a long simulation time and the assumption of having the same effect due to a significant number of examples, many authors do not choose the cross-validation method when using deep learning. On the other hand, cross-validation of event-based apnea detection techniques is frequently used without ensuring subject independent (or this information was not mention specifically in the paper), which is essential to assess the generalization capability of the model. Some authors used dataset balancing methods or specific parameters to solve the class imbalance problem. It was also not clear for some works if the test dataset was balanced or not, which should not be done since it will change the natural distribution of data and, consequently, derail the generalization of the model. To have a fair test, a form of cross-validation with subject independence could be suggested as a good choice for future research.

There are two main classification strategies; event-by-event or global classification. Most of the works concentrated on event-by-event classification and eight works used global classification considering OSA severity classification. However, it is possible to do a global classification from event-by-event classification methods by using a threshold approach as indicated by Pathinarupothi et al. [33]. This observation is considered extremely relevant for further research since it will allow the methods to be used for clinical diagnosis.

## Figures and Tables

**Table 1:** Summary of the database information: The database, year of publication, number of subjects, used signals, window size and type of classifiers (A = apnea, H = hypopnea, N = normal, S = severity, O = obstructive, G = global or obstructive sleep apnea (OSA) severity) used by selected papers (according to year).

Paper	Year	Database	Recordings	Sensors/Signals	Window Size (Seconds)	Classification Type
[35]	2008	Apnea-ECG Database (AED) [29]	70	[Heart rate variability (HRV)- electrocardiogram (ECG)]	60	A/N
[60]	2017	Multi-Ethnic Study of Atherosclerosis (MESA)	100	[Nasal airflow]	30	OA/N
[47]	2017	AED [29]	8	[Blood oxygen saturation index (SpO_2_)]	60	OA/N
		University College Dublin Sleep Apnea Database (UCD) [61]	25	[SpO_2_]	60	A/N
[34]	2017	AED [29]	35	[Instantaneous heart rates (IHR)-ECG]	60	G
[33]	2017	AED [29]	35	[IHR-ECG]	60	OA/N, G
		AED [29]	8	[SpO_2_]	60	OA/N, G
[62]	2017	AED [29]	35	[ECG inter-beat intervals (RR-ECG)]	-	OA/N
[37]	2018	AED [29]	35	[ECG]	60	OA/N
[59]	2018	MESA [39]	1507	[Nasal airflow]	30	A/H/N
[52]	2018	Seoul National University Bundang Hospital (SNUBH) [52]	120	[Breathing sounds]	5	G
[46]	2018	Sleep Center of Samsung Medical Center, Seoul, Korea (SCSMC82) [46]	82	[ECG]	10	OA/N
[48]	2018	UCD [61]	23	[SpO_2_, oronasal airflow, and ribcage and abdomen movements]	1	OAH/N
[53]	2018	MESA [39]	1507	[Nasal airflow, Abdominal and thoracic plethysmography]	30	OA/H/N
[36]	2018	AED [29]	35	[HRV ECG]	60	OA/N
[51]	2018	Seoul National University Hospital (SNUH) [51],	179	[Nasal pressure]	10	AH/N, G
		MESA [39]	50	[Nasal pressure]	10	AH/N, G
[38]	2018	Osteoporotic Fractures in Men Study (MrOS) (Visit 1) [40]	545	[ECG]	15	G
[57]	2018	MrOS (Visit 2) [40]	520	[Airflow]	-	G
[49]	2018	Massachusetts General Hospital (MGH)	10 000	[Airflow, respiration (chest and abdomen belts), SpO_2_]	1	G
		Sleep Heart Health Study (SHHS) [50]	5804	[Airflow, respiration (chest and abdomen belts), SpO_2_]	1	G
[55]	2018	SHHS-1 [54]	2100	[Respiratory signals (chest and abdomen belts), ECG derived respiration (EDR)]	30	A/N
[43]	2018	SCSMC86 [43]	86	[ECG]	10	OA/H/N
[45]	2018	SCSMC92	92	[ECG]	10	A/H/N, AH/N
[32]	2018	AED [29]	70	[RR–ECG]	60	OAH/N, G

**Table 2:** Performance of the different works.

Paper	Classifier Type	Sen/Recall (%)	Spc (%)	Acc (%)	Others
[57]	Multiple hidden layers neural network (MHLNN) (Apnea Hypopnea Index, AHI 5)	80.47 (G)	86.35 (G)	83.46 (G)	-
	MHLNN (AHI 15)	85.56 (G)	86.96 (G)	85.39 (G)	-
	MHLNN (AHI 30)	93.06 (G)	90.23 (G)	92.69 (G)	-
[36]	MHLNN	-	-	68.37	-
[52]	MHLNN	-	-	75 (G)	-
[32]	Stacked autoencoder (SAE)	88.9	88.4	83.8	Area under the receiver operating characteristic curve (AUC) 0.86.9
	SAE	100 (G)	100 (G)	100 (G)	
[47]	Deep belief networks (DBN), (UCD)	60.36	91.71	85.26	Combined objective (CO) 79.1
	DBN (AED)	78.75	95.89	97.64	-
[43] *	Convolution neural network (CNN)1D	87	87	90.8	Positive predictive value, $\left(\mathrm{PPV}\right)87\%,F1_{w}\;87.0$
[46] *	CNN1D	96	96	96	$F1_{w}\;$0.96
[37]	CNN1D	97.82	99.20	98.91	PPV 99.06%, negative predictive value (NPV) 98.14%
[51]	CNN1D	81.1	98.5	96.6	PPV 87%, NPV 97.7%
	CNN1D (AHI 5)	100 (G)	84.6 (G)	96.2 (G)	PPV 95.1%, NPV 100%, $F_{1}$ 0.98 (G)
	CNN1D (AHI 15)	98.1 (G)	86.5 (G)	92.3 (G)	PPV 87.9%, NPV 97.8%, $F_{1}$ 0.93 (G)
	CNN1D (AHI 30)	96.2 (G)	96.2 (G)	96.2 (G)	PPV 89.3%, NPV 98.7%, $F_{1}$ 0.93 (G)
[60]	CNN1D	74.70	-	74.70	PPV 74.50%
[53]	CNN1D-3ch	83.4	-	83.5	PPV 83.4%, $F_{1}$ 83.4
[59]	CNN1D	77.6	-	77.6	PPV 77.4%, $F_{1}$ 77.5
	CNN2D	79.7	-	79.8	PPV 79.8%, $F_{1}$ 79.7
[48]	CNN2D		-	79.6	-
[33]	Long short-term memory (LSTM), (SpO_2_)	92.9	-	95.5	AUC 0.98, PPV 99.2%
	LSTM (IHR)	99.4	-	89.0	AUC 0.99%, PPV 82.4%
	LSTM (SpO_2_ + IHR)	84.7	-	92.1	AUC 0.99%, PPV 99.5%
	LSTM (IHR)	99.4 (G)			
[34]	LSTM (IHR)	-	-	100 (G)	$F_{1}$ 1 (G)
[62]	LSTM	-	-	97.08	-
[35]	FLSTM	85.5	80.1	82.1	-
[55]	FLSTM (abdores)	57.9	73.9	71.1	AUC 71.5, PPV 33.0%
	LSTM (abdores)	62.3	80.3	77.2	AUC 77.5, PPV 39.9%
	FLSTM (thorres)	62.9	77.2	74.7	AUC 76.9, PPV 36.8%
	LSTM (thorres)	67.8	76.5	75	AUC 79.7, PPV 37.7%
	FLSTM (EDR)	48.8	60.8	58.7	AUC 57.6, PPV 21.1%
	LSTM (EDR)	52.1	61.8	60.1	AUC 58.8, PPV 22.1%
[45]	LSTM	98	98	98.5	$F_{1w}$ 98.0
	Gated recurrent unit (GRU)	99	99	99.0	$F_{1w}$ 99.0
[49]	Recurrent and convolutional neural networks (RCNN), (MGH)	-	-	88.2 (G)	-
[38]	CNN1D-LSTM- MHLNN	77.60 (G)	80.10 (G)	79.45 (G)	$F_{1}$ 79.09 (G)

* The authors used an alternative definition of true positive (detection of normal events) compared with the definition provided by Baratloo et al. [88]. Therefore, in this table for binary classifier comparing with other authors their Sen could be treated as Spc and vice versa. If nothing is indicated in the paper, then an assumption was made that the authors did use the definition provided in Baratloo et al.

## Appendix A

**Table A1:** Abbreviations and Acronyms used in this work. (according to alphabetic order Top- Bottom, Left-Right).

Abbreviation and Acronyms	Full Form	Abbreviation and Acronyms	Full Form
AASM	American Academy of Sleep Medicine	LSTM	Long Short-Term Memory
Acc	Accuracy	maxpooling	Maximum Pooling
AE	Autoencoder	MESA	Multi-Ethnic Study of Atherosclerosis
AED	Apnea-ECG database	MGH	Massachusetts General Hospital
AF	Air Flow	MHLNN	Multiple hidden layers neural network
AHI	Apnea hyperpnea Index	mRMR	Minimum Redundancy Maximum Relevance
ANN	Artificial Neural Network	MrOS	Osteoporotic Fractures in Men Study
AUC	Area under ROC curve	NPV	Negative Predictive Value
bpm	Beats Per Minutes	NSRR	National Sleep Research Resource
CNN	Convolution Neural Network	OSA	Obstructive Sleep Apnea
CO	Combined Objective	OSAH	Obstructive Sleep Apnea Hypopnea
CWT	Continuous Wavelet Transform	SpO_2_	Blood Oxygen Saturation Index
DAE	Deep Autoencoder	Spc	Specificity
DBN	Deep Belief Network	PPV	Precision or Positive Predictive Value
DL	Deep Learning	PSG	Polysomnography
DNN	Deep Neural Network	RBM	Restricted Boltzmann Machines
DVNN	Deep Vanilla Neural Network	RCNN	Combined Deep Recurrent and Convolutional Neural Networks
EA	Evolutionary Algorithms	ReLU	Rectified Linear Unit
ECG	Electrocardiography	RF	Random Forest
EDR	ECG derived respiration	RNN	recurrent neural network
EEG	Electroencephalogram	RR-ECG	R to R interval from ECG
EMG	Electromyography	SAE	Stacked Autoencoder
EOG	Electrooculogram	SCSMC	Sleep Center of Samsung Medical Center
$F_{1}$	$F_{1}$ score	Sen	Recall or Sensitivity
$F_{1w}$	Weighted $F_{1}$ score	SFS	Sequential Forward Selection
FP	False Positive	SHHS	Sleep Heart Health Study
FLSTM	LSTM with feature inputs	SNUBH	Seoul National University Bundang Hospital
GA	Genetic Algorithm	SNUH	Seoul National University Hospital
HRV	Heart Rate Variability	SpAE	Sparse Autoencoder
Hz	Hertz	Spc	Specificity
IHR	Instantaneous Heart Rates	SVM	Support Vector Machine
IIR	Infinite Impulse Response	TN	True Negative
kNN	k-nearest neighbor	TP	True Positive
LDA	Linear Discriminant Analysis	UCD	St. Vincent’s University Hospital/University College Dublin Sleep Apnea Database
LR	Logistic Regression		

## References

[1] Sateia M.J. (2014). International Classification of Sleep Disorders-Third Edition (ICSD-3). Chest.

[2] Zhang J., Zhang Q., Wang Y., Qiu C. A Real-time auto-adjustable smart pillow system for sleep apnea detection and treatment. Proceedings of the 12th International Conference on Information Processing in Sensor Networks (IPSN).

[3] Young T., Palta M., Dempsey J., Skatrud J., Weber S., Badr S. (1993). The occurrence of sleep-disordered breathing among middle-aged adults. N. Engl. J. Med..

[4] Young T., Evans L., Finn L., Palta M. (1997). Estimation of the clinically diagnosed proportion of sleep apnea syndrome in middle-aged men and women. Sleep.

[5] Gislason T., Benediktsdóttir B. (1995). Snoring, Apneic Episodes, and Nocturnal Hypoxemia Among Children 6 Months to 6 Years Old. Chest.

[6] Ancoli-Israel S., DuHamel E.R., Stepnowsky C., Engler R., Cohen-Zion M., Marler M. (2003). The relationship between congestive heart failure, sleep apnea, and mortality in older men. Chest.

[7] Vgontzas A.N., Papanicolaou D.A., Bixler E.O., Hopper K., Lotsikas A., Lin H.-M., Kales A., Chrousos G.P. (2000). Sleep Apnea and Daytime Sleepiness and Fatigue: Relation to Visceral Obesity, Insulin Resistance, and Hypercytokinemia. J. Clin. Endocrinol. Metab..

[8] Doumit J., Prasad B. (2016). Sleep Apnea in Type 2 Diabetes. Diabetes Spectr..

[9] Bsoul M., Minn H., Tamil L. (2011). Apnea MedAssist: Real-time Sleep Apnea Monitor Using Single-Lead ECG. IEEE Trans. Inf. Technol. Biomed..

[10] De Chazal P., Penzel T., Heneghan C. (2004). Automated Detection of Obstructive Sleep Apnoea at Different Time Scales using the Electrocardiogram. Physiol. Meas..

[11] Agarwal R., Gotman J. (2001). Computer-Assisted Sleep Staging. IEEE Trans. Biomed. Eng..

[12] Hillman D.R., Murphy A.S., Pezzullo L. (2006). The Economic Cost of Sleep Disorders. Sleep.

[13] Alghanim N., Comondore V.R., Fleetham J., Marra C.A., Ayas N.T. (2008). The Economic Impact of Obstructive Sleep Apnea. Lung.

[14] Khandoker A.H., Gubbi J., Palaniswami M. (2009). Automated Scoring of Obstructive Sleep Apnea and Hypopnea Events Using Short-Term Electrocardiogram Recordings. IEEE Trans. Inf. Technol. Biomed..

[15] Mendez M.O., Corthout J., Van Huffel S., Matteucci M., Penzel T., Cerutti S., Bianchi A.M. (2010). Automatic screening of obstructive sleep apnea from the ECG based on empirical mode decomposition and wavelet analysis. Physiol. Meas..

[16] Mostafa S.S., Morgado-Dias F., Ravelo-García A.G. (2018). Comparison of SFS and mRMR for oximetry feature selection in obstructive sleep apnea detection. Neural Comput. Appl..

[17] Al-Angari H.M., Sahakian A.V. (2012). Automated Recognition of Obstructive Sleep Apnea Syndrome Using Support Vector Machine Classifier. IEEE Trans. Inf. Technol. Biomed..

[18] Álvarez-Estévez D., Moret-Bonillo V. (2009). Fuzzy reasoning used to detect apneic events in the sleep apnea-hypopnea syndrome. Expert Syst. Appl..

[19] Lee S., Urtnasan E., Lee K.-J. (2017). Design of a Fast Learning Classifier for Sleep Apnea Database based on Fuzzy SVM. Int. J. Fuzzy Log. Intell. Syst..

[20] Almazaydeh L., Faezipour M., Elleithy K. (2012). A Neural Network System for Detection of Obstructive Sleep Apnea Through SpO2 Signal Features. Int. J. Adv. Comput. Sci. Appl..

[21] Mostafa S.S., Carvalho J.P., Morgado-Dias F., Ravelo-García A. Optimization of sleep apnea detection using SpO2 and ANN. Proceedings of the XXVI International Conference on Information, Communication and Automation Technologies (ICAT).

[22] Ravelo-García A., Kraemer J., Navarro-Mesa J., Hernández-Pérez E., Navarro-Esteva J., Juliá-Serdá G., Penzel T., Wessel N. (2015). Oxygen Saturation and RR Intervals Feature Selection for Sleep Apnea Detection. Entropy.

[23] Cover T.M. (1974). The Best Two Independent Measurements Are Not the Two Best. IEEE Trans. Syst. Man Cybern..

[24] Mendez M.O., Bianchi A.M., Matteucci M., Cerutti S., Penzel T. (2009). Sleep Apnea Screening by Autoregressive Models from a Single ECG Lead. IEEE Trans. Biomed. Eng..

[25] Isa S.M., Fanany M.I., Jatmiko W., Arymurthy A.M. Sleep apnea detection from ECG signal: Analysis on optimal features, principal components, and nonlinearity. Proceedings of the IEEE 5th International Conference on Bioinformatics and Biomedical Engineering.

[26] Mendonça F., Mostafa S.S., Ravelo-García A.G., Morgado-Dias F., Penzel T. (2018). Devices for Home Detection of Obstructive Sleep Apnea: A Review. Sleep Med. Rev..

[27] Mendonca F., Mostafa S.S., Ravelo-Garcia A.G., Morgado-Dias F., Penzel T. (2019). A Review of Obstructive Sleep Apnea Detection Approaches. IEEE J. Biomed. Health Inform..

[28] Jayaraj R., Mohan J., Kanagasabai A. (2017). A Review on Detection and Treatment Methods of Sleep Apnea. J. Clin. Diagn. Res..

[29] Penzel T., Moody G., Mark R., Goldberger A., Peter J. (2000). The apnea-ECG database. Proceedings of the Computers in Cardiology.

[30] PhysioNet. www.physionet.org.

[31] Goldberger A.L., Amaral L.A., Glass L., Hausdorff J.M., Ivanov P.C., Mark R.G., Mietus J.E., Moody G.B., Peng C.K., Stanley H.E. (2000). PhysioBank, PhysioToolkit, and PhysioNet: Components of a New Research Resource for Complex Physiologic Signals. Circulation.

[32] Li K., Pan W., Li Y., Jiang Q., Liu G. (2018). A method to detect sleep apnea based on deep neural network and hidden Markov model using single-lead ECG signal. Neurocomputing.

[33] Pathinarupothi R.K., Rangan E.S., Gopalakrishnan E.A., Vinaykumar R., Soman K.P. (2017). Single sensor techniques for sleep apnea diagnosis using deep learning. Proceedings of the IEEE International Conference on Healthcare Informatics (ICHI).

[34] Pathinarupothi R.K., Vinaykumar R., Rangan E., Gopalakrishnan E., Soman K.P. (2017). Instantaneous heart rate as a robust feature for sleep apnea severity detection using deep learning. Proceedings of the IEEE EMBS International Conference on Biomedical & Health Informatics (BHI).

[35] Novak D., Mucha K., Al-Ani T. Long Short-Term Memory for apnea detection based on heart rate variability. Proceedings of the 30th Annual International Conference of the IEEE Engineering in Medicine and Biology Society.

[36] De Falco I., De Pietro G., Sannino G., Scafuri U., Tarantino E., Della Cioppa A., Trunfio G.A. (2018). Deep neural network hyper-parameter setting for classification of obstructive sleep apnea episodes. Proceedings of the 2018 IEEE Symposium on Computers and Communications (ISCC).

[37] Dey D., Chaudhuri S., Munshi S. (2018). Obstructive sleep apnoea detection using convolutional neural network based deep learning framework. Biomed. Eng. Lett..

[38] Banluesombatkul N., Rakthanmanon T., Wilaiprasitporn T. Single Channel ECG for Obstructive Sleep Apnea Severity Detection using a Deep Learning Approach. Proceedings of the TENCON 2018—2018 IEEE Region 10 Conference.

[39] Dean D.A., Goldberger A.L., Mueller R., Kim M., Rueschman M., Mobley D., Sahoo S.S., Jayapandian C.P., Cui L., Morrical M.G. (2016). Scaling Up Scientific Discovery in Sleep Medicine: The National Sleep Research Resource. Sleep.

[40] Blank J.B., Cawthon P.M., Carrion-Petersen M.L., Harper L., Johnson J.P., Mitson E., Delay R.R. (2005). Overview of recruitment for the osteoporotic fractures in men study (MrOS). Contemp. Clin. Trials.

[41] Orwoll E., Blank J.B., Barrett-Connor E., Cauley J., Cummings S., Ensrud K., Lewis C., Cawthon P.M., Marcus R., Marshall L.M. (2005). Design and baseline characteristics of the osteoporotic fractures in men (MrOS) study--a large observational study of the determinants of fracture in older men. Contemp. Clin. Trials.

[42] Blackwell T., Yaffe K., Ancoli-Israel S., Redline S., Ensrud K., Stefanick M., Laffan A., Stone K. (2011). Associations between sleep architecture and sleep-disordered breathing and cognition in older community-dwelling men: The Osteoporotic Fractures in Men Sleep Study. J. Am. Geriatr. Soc..

[43] Urtnasan E., Park J.-U., Lee K.-J. (2018). Multiclass classification of obstructive sleep apnea/hypopnea based on a convolutional neural network from a single-lead electrocardiogram. Physiol. Meas..

[44] Berry B.R., Brooks R., Gamaldo E.C., Harding M.S., Marcus C., Vaughn B. (2012). AASM Manual for the Scoring of Sleep and Associated Events. Rules, Terminology and Technical Specifications.

[45] Urtnasan E., Park J.U., Lee K.J. (2018). Automatic detection of sleep-disordered breathing events using recurrent neural networks from an electrocardiogram signal. Neural Comput. Appl..

[46] Urtnasan E., Park J., Joo E., Lee K. (2018). Automated Detection of Obstructive Sleep Apnea Events from a Single-Lead Electrocardiogram Using a Convolutional Neural Network. J. Med. Syst..

[47] Mostafa S.S., Mendonça F., Morgado-Dias F., Ravelo-García A. (2017). SpO2 based sleep apnea detection using deep learning. Proceedings of the 2017 IEEE 21st International Conference on Intelligent Engineering Systems (INES).

[48] Cen L., Yu Z.L., Kluge T., Ser W. (2018). Automatic system for obstructive sleep apnea events detection using convolutional neural network. Proceedings of the 40th Annual International Conference of the IEEE Engineering in Medicine and Biology Society (EMBC).

[49] Biswal S., Sun H., Goparaju B., Westover M.B., Sun J., Bianchi M.T. (2018). Expert-level sleep scoring with deep neural networks. J. Am. Med. Informatics Assoc..

[50] Sleep Heart Health Study. https://sleepdata.org/datasets/shhs.

[51] Choi S.H., Yoon H., Kim H.S., Kim H.B., Kwon H.B., Oh S.M., Lee Y.J., Park K.S. (2018). Real-time apnea-hypopnea event detection during sleep by convolutional neural networks. Comput. Biol. Med..

[52] Kim T., Kim J.-W., Lee K. (2018). Detection of sleep disordered breathing severity using acoustic biomarker and machine learning techniques. Biomed. Eng. Online.

[53] Haidar R., McCloskey S., Koprinska I., Jeffries B. (2018). Convolutional neural networks on multiple respiratory channels to detect hypopnea and obstructive apnea events. Proceedings of the 2018 International Joint Conference on Neural Networks (IJCNN).

[54] Quan S.F., Howard B.V., Iber C., Kiley J.P., Nieto F.J., O’Connor G.T., Rapoport D.M., Redline S., Robbins J., Samet J.M. (1997). The Sleep Heart Health Study: Design, rationale, and methods. Sleep.

[55] Van Steenkiste T., Groenendaal W., Deschrijver D., Dhaene T. (2018). Automated Sleep Apnea Detection in Raw Respiratory Signals using Long Short-Term Memory Neural Networks. IEEE J. Biomed. Heal. Informatics.

[56] Technical Notes on SHHS1. https://www.sleepdata.org/datasets/shhs/pages/08-equipment-shhs1.md.

[57] Lakhan P., Ditthapron A., Banluesombatkul N., Wilaiprasitporn T. Deep neural networks with weighted averaged overnight airflow features for sleep apnea-hypopnea severity classification. Proceedings of the TENCON, IEEE Region 10 International Conference.

[58] Lee-Chiong T.L. (2008). Sleep Medicine: Essentials and Review.

[59] McCloskey S., Haidar R., Koprinska I., Jeffries B. (2018). Detecting hypopnea and obstructive apnea events using convolutional neural networks on wavelet spectrograms of nasal airflow. Proceedings of the Pacific-Asia Conference on Knowledge Discovery and Data Mining (PAKDD).

[60] Haidar R., Koprinska I., Jeffries B. Sleep apnea event detection from nasal airflow using convolutional neural networks. Proceedings of the International Conference on Neural Information Processing (ICONIP).

[61] St. Vincent’s University Hospital/University College Dublin Sleep Apnea Database. https://physionet.org/pn3/ucddb/.

[62] Cheng M., Sori W.J., Jiang F., Khan A., Liu S. (2017). Recurrent neural network based classification of ECG signal features for obstruction of sleep apnea detection. Proceedings of the 2017 IEEE International Conference on Computational Science and Engineering (CSE) and IEEE International Conference on Embedded and Ubiquitous Computing (EUC).

[63] Boll S. (1979). Suppression of acoustic noise in speech using spectral subtraction. IEEE Trans. Acoust..

[64] Kim J., Kim T., Lee D., Kim J.-W., Lee K. (2017). Exploiting temporal and nonstationary features in breathing sound analysis for multiple obstructive sleep apnea severity classification. Biomed. Eng. Online.

[65] Van Steenkiste T., Groenendaal W., Ruyssinck J., Dreesen P., Klerkx S., Smeets C., de Francisco R., Deschrijver D., Dhaene T. (2018). Systematic comparison of respiratory signals for the automated detection of sleep apnea. Proceedings of the 40th Annual International Conference of the IEEE Engineering in Medicine and Biology Society (EMBC).

[66] Tian J.Y., Liu J.Q. (2005). Apnea detection based on time delay neural network. Proceedings of the 2005 IEEE Engineering in Medicine and Biology 27th Annual Conference.

[67] Pan J., Tompkins W.J. (1985). A Real-Time QRS Detection Algorithm. IEEE Trans. Biomed. Eng..

[68] Chen L., Zhang X., Song C. (2015). An Automatic Screening Approach for Obstructive Sleep Apnea Diagnosis Based on Single-Lead Electrocardiogram. IEEE Trans. Autom. Sci. Eng..

[69] Software for Viewing, Analyzing, and Creating Recordings of Physiologic Signals. https://physionet.org/physiotools/wfdb.shtml.

[70] Niskanen J.-P., Tarvainen M.P., Ranta-aho P.O., Karjalainen P.A. (2004). Software for advanced HRV analysis. Comput. Methods Programs Biomed..

[71] Haykin S. (2001). Neural Networks: A Comprehnsive Foundation.

[72] Goodfellow I., Bengio Y., Courville A. (2016). Deep Learning.

[73] Olshausen B.A., Field D.J. (1997). Sparse coding with an overcomplete basis set: A strategy employed by V1?. Vision Res..

[74] Salakhutdinov R., Murray I. (2008). On the quantitative analysis of deep belief networks. Proceedings of the 25th International Conference on Machine learning—ICML ’08.

[75] Ren J.S.J., Xu L. On vectorization of deep convolutional neural networks for vision tasks. Proceedings of the 29th AAAI Conference on Artificial Intelligence.

[76] Stutz D. (2016). Understanding Convolutional Neural Networks. Nips.

[77] Nagi J., Ducatelle F. Max-pooling convolutional neural networks for vision-based hand gesture recognition. Proceedings of the IEEE International Conference on Signal and Image Processing Applications (ICSIPA).

[78] Baptista D., Mostafa S., Pereira L., Sousa L., Morgado-Dias F., Baptista D., Mostafa S.S., Pereira L., Sousa L., Morgado-Dias F. (2018). Implementation Strategy of Convolution Neural Networks on Field Programmable Gate Arrays for Appliance Classification Using the Voltage and Current (V-I) Trajectory. Energies.

[79] Memisevic R., Zach C., Hinton G.E., Pollefeys M. Gated softmax classification. Proceedings of the Advances in Neural Information Processing Systems 23 (NIPS 2010).

[80] Ioffe S., Szegedy C. Batch normalization: Accelerating deep network training by reducing internal covariate shift. Proceedings of the ICML’15 32nd International Conference on International Conference on Machine Learning.

[81] Srivastava N., Hinton G., Krizhevsky A., Sutskever I., Salakhutdinov R. (2014). Dropout: A Simple Way to Prevent Neural Networks from Overfitting. J. Mach. Learn. Res..

[82] Krizhevsky A., Sutskever I., Hinton G.E. (2017). ImageNet Classification with Deep Convolutional Neural Networks. Commun. ACM.

[83] Gao Y., Glowacka D. Deep Gate Recurrent Neural Network. Proceedings of the Asian Conference on Machine Learning.

[84] Hochreiter S., Urgen Schmidhuber J. (1997). Long short-term memory. Neural Comput..

[85] Zhang H., Li J., Ji Y., Yue H. (2017). Understanding Subtitles by Character-Level Sequence-to-Sequence Learning. IEEE Trans. Ind. Informatics.

[86] Chung J., Gulcehre C., Cho K., Bengio Y. (2014). Empirical Evaluation of Gated Recurrent Neural Networks on Sequence Modeling. arXiv.

[87] Baratloo A., Hosseini M., Negida A., El Ashal G. (2015). Part 1: Simple Definition and Calculation of Accuracy, Sensitivity and Specificity. Emergency (Iran).

[88] Fawcett T. (2004). ROC Graphs: Notes and Practical Considerations for Data Mining Researchers. Hp L-2003-4. Mach. Learn..

[89] Vluymans S. (2019). Learning from imbalanced data. Stud. Comput. Intell..

[90] Wallace B.C., Small K., Brodley C.E., Trikalinos T.A. Class imbalance, redux. Proceedings of the IEEE 11th International Conference on Data Mining.

[91] Johnson J.M., Khoshgoftaar T.M. (2019). Survey on deep learning with class imbalance. J. Big Data.

[92] Song C., Liu K., Zhang X., Chen L., Xian X. (2016). An Obstructive Sleep Apnea Detection Approach Using a Discriminative Hidden Markov Model from ECG Signals. IEEE Trans. Biomed. Eng..

[93] Nguyen H.D., Wilkins B.A., Cheng Q., Benjamin B.A. (2014). An Online Sleep Apnea Detection Method Based on Recurrence Quantification Analysis. IEEE J. Biomed. Health Inform..

